# Tuning Redox Potential of Anthraquinone-2-Sulfonate (AQS) by Chemical Modification to Facilitate Electron Transfer From Electrodes in *Shewanella oneidensis*

**DOI:** 10.3389/fbioe.2021.705414

**Published:** 2021-08-10

**Authors:** Ning Xu, Tai-Lin Wang, Wen-Jie Li, Yan Wang, Jie-Jie Chen, Jun Liu

**Affiliations:** ^1^ Tianjin Institute of Industrial Biotechnology, Chinese Academy of Sciences, Tianjin, China; ^2^ Key Laboratory of Systems Microbial Biotechnology, Chinese Academy of Sciences, Tianjin, China; ^3^ College of Life Sciences, University of Chinese Academy of Sciences, Beijing, China; ^4^ Department of Environmental Science and Engineering, University of Science and Technology of China, Hefei, China

**Keywords:** bioelectrochemical systems, electron shuttles, rational design, coulombic efficiency, Mtr pathway

## Abstract

Bioelectrochemical systems (BESs) are emerging as attractive routes for sustainable energy generation, environmental remediation, bio-based chemical production and beyond. Electron shuttles (ESs) can be reversibly oxidized and reduced among multiple redox reactions, thereby assisting extracellular electron transfer (EET) process in BESs. Here, we explored the effects of 14 ESs on EET in *Shewanella oneidensis* MR-1, and found that anthraquinone-2-sulfonate (AQS) led to the highest cathodic current density, total charge production and reduction product formation. Subsequently, we showed that the introduction of -OH or -NH_2_ group into AQS at position one obviously affected redox potentials. The AQS-1-NH_2_ exhibited a lower redox potential and a higher Coulombic efficiency compared to AQS, revealing that the ESs with a more negative potential are conducive to minimize energy losses and improve the reduction of electron acceptor. Additionally, the cytochromes MtrA and MtrB were required for optimal AQS-mediated EET of *S. oneidensis* MR-1. This study will provide new clues for rational design of efficient ESs in microbial electrosynthesis.

## Introduction

Energy crisis and environmental pollution are one of the major global issues that the world is facing today. The bioelectrochemical system (BES), including microbial fuel cell (MFC) and microbial electrosynthesis (MES), has been regarded as a promising technology for energy generation, resource recovery, environmental remediation, and chemical production (Bajracharya et al., 2016; Chen et al., 2020a; Naha et al., 2020; Rout et al., 2020). In a MFC system, the microorganisms transfer electrons to a solid-substrate for the generation of electrical power coupled to the oxidation of organic or inorganic matters in the wastewater (Santoro et al., 2017). On the other hand, MES reverses the direction of electron transfer from a cathode to microbes for the microbial production of value-added fuels or chemicals (Rabaey and Rozendal, 2010; Das et al., 2020). In addition, MES enables the biocatalysts to use a variety of clean and renewable electricity-sources, including solar, wind, geothermal, biomass, hydropower and surpluses electricity from the power grid (Zhang, 2015). To date, MES has been applied successfully for the production of valuable fuels and commodity chemicals by the direct electrochemical reduction of CO_2_, such as methane, formate, acetate, ethanol, butyrate and other higher biofuels, which offers an environmentally friendly alternative to the products typically derived from fossil (Bian et al., 2020; Prévoteau et al., 2020).

Although MES represents a promising platform for the renewable energy storage and value-added chemical production, it is currently limited by low electron transfer rates from a cathode to microbes (Kracke et al., 2015; Chen et al., 2020b; Zhao et al., 2020). Electron shuttles (ESs) provide an effective conduit for the extracellular electron transfer (EET) process from the electrode to microbial cells. ESs, also known as redox mediators, were typically chosen based on their midpoint potentials between the electron acceptor and electron donor (Watanabe et al., 2009; Li et al., 2020). The use of ESs offers a possibility to control the amount of available redox equivalents and to enhance the EET process of microbes, whereas the possible toxic or unstable behaviors of these shuttles impede their applications in some cases (Hongo and Iwahara, 1979; Light et al., 2018). The underlying mechanisms for electron transfer between electrodes and microbes were majorly investigated in a model dissimilatory metal reducing bacterium (DMRB) *Shewanella oneidensis* MR-1 (Kracke et al., 2015; Kumar et al., 2017; Rowe et al., 2018). This strain was reported to use a broad spectrum of terminal electron acceptors, such as heavy metal minerals, fumarate, dimethyl sulfoxide, nitrate and thiosulfate (Ikeda et al., 2021). *S. oneidensis* MR-1 employs several different strategies to capture electrons from the cathode, including cytochromes, nanowires and extracellular ESs (Tremblay and Zhang, 2015). Nowadays, many ESs have been found to improve the electron transport capacity from a cathode to microbes, such as neutral red (NR), riboflavin (RF), anthraquinone-2-sulfonate (AQS) and methyl viologen (MV) (Choi et al., 2012; Harrington et al., 2015a; Tokunou et al., 2016; Xafenias et al., 2016).

The pleiotropic effects of different ESs on electrical conductivity in MES were previously reported in the literature. For example, Tokunou et al. (2016) reported that two flavin-like polycyclic molecules, safranin and α-AQS, showed different cathodic EET kinetics and current production with riboflavin in an electrode-immobilized biofilm reactor. In addition, Harrington et al. (2015a) also found that NR-mediated microbial electrosynthesis was shown to have multifarious effects on three different bacteria. Hence, the choice of a proper electron mediator is of great significance for improving the performance characteristics of MES system. The redox potential reflects an inherent property of ESs and is a critical parameter associated with the ESs-mediated EET process (Van der Zee and Cervantes, 2009). The changes in molecular structure of ESs by the substituent group typically affect their redox potentials, thereby influencing the electron transfer (Rauschnot et al., 2009; Chen et al., 2013; Er et al., 2015). In addition, although several efforts have focused on the direct electron uptake mechanisms from cathodes to microbes in recent years (Kracke et al., 2015; Rowe et al., 2018; Zhao et al., 2020), little is known about the underlying molecular mechanisms of ESs-mediated EET process in MES.

In this study, 14 different types of ESs were chosen to investigate their effects on the cathodic EET capacity of *S. oneidensis* MR-1. Subsequently, the most promising ES was rationally designed and chemically modified to fine-tuning redox potentials in order to improve the EET capacity. Meanwhile, the molecular mechanisms underlying the ESs-mediated EET process of *S. oneidensis* MR-1 were also explored. These findings will provide a new mechanistic understanding of ESs-mediated EET capacity, and offer useful information for designing more efficient ESs.

## Materials and Methods

### Strain Construction and Culture Conditions

Bacterial strains and plasmids used in this study are listed in Supplementary Table S1. The primers used in this study are listed in Supplementary Table S2. *E. coli* DH5α was used as host cells for general cloning, and *E. coli* WM3064 was used as a conjugation donor strain. *S. oneidensis* MR-1 was used as the wild-type strain for gene disruption. The corresponding mutants of *S. oneidensis* were generated by an efficient conjugation-based in-frame deletion mutagenesis with pHG1.0 suicide plasmid as described previously (Yin et al., 2018). For routine purposes, *S. oneidensis* strains were grown in LB medium (0.5% yeast extract, 1% tryptone, 1% NaCl) at 32°C with shaking.

### Chemicals or Reagents

Neutral red (NR), methyl viologen dichloride hydrate (MV), potassium hexacyanoferrate (PH), 2,6-dichlorophenolindophenol sodium salt hydrate (DCPIP), thionin acetate salt (LV), 2,3,5-triphenyltetrazoliun chloride (TTC), alizarin red (AR), riboflavin (RF), humic acid (HA), benzyl viologen dichloride (BV), 2,6-di-tert-butyl-1,4-benzo-quinone (DTBBQ), phenazine methosulfate (PMS), methylene blue (MB), anthraquinone-2-sulfonate (AQS), and anthraquinone-2,6-disulfonate (AQDS) were purchased from Sigma-Aldrich (St. Louis, MO, United States). AQS-1-NH_2_ and AQS-1-OH agents were obtained by chemical synthesis from Accela ChemBio (Shanghai, China).

### Toxicity Assay

A single colony from the freshly streaked agar plate was transferred to LB medium, and pre-grown for 16 h at 32°C prior to growth experiments. The cultures were harvested, washed, and re-suspended in the fresh LB medium with an initial cell density (OD_600_) of 0.05. The tested ESs at five different concentrations (0, 0.01, 0.05, 0.1, 0.5 mM) were added to the medium. 200 μl of cell suspensions was seeded in triplicate into 96-well culture plates, and incubated at 32°C with shaking at 800 rpm in a Microtron shaking incubator (INFORS-HT, Switzerland) (Lam et al., 2018). Cell growth was monitored by measuring the optical density at 600 nm after 8 h of incubation. Results are representative of at least two independent experiments performed in triplicate and are expressed as mean ± standard deviation (SD).

### Microbial Electrosynthesis Reactor Setup

Double-chamber BESs with a working volume of 135 ml were used in this study (Supplementary Figure S1A) (Xafenias et al., 2015; Li et al., 2017). Carbon cloth substrates with a surface area of 4 cm^2^ were used as the working electrode. Ag/AgCl electrodes (in a 3M KCl solution) and Pt wires (0.1 mm diameter, 2 cm in length immersed into the solution) were used as the reference and counter electrodes, respectively. The potential of Ag/AgCl (3M KCl) reference electrode against standard hydrogen electrode (SHE) is approximately +0.210 V. The working and reference electrodes were placed in a cathode chamber, and the counter electrode was placed in an anode chamber. These two chambers were separated by a pretreated 4 cm^2^ Nafion-117 proton exchange membrane (Dupont Co., United States). The electrolyte in chambers was composed of 95% M9 buffer (17.8 gL^−1^ Na_2_HPO_4_⋅12H_2_O, 3.0 gL^−1^ KH_2_PO_4_, 1.0 gL^−1^ NH_4_Cl, 0.5 gL^−1^ NaCl, 1.0 mM MgSO_4_⋅7H_2_O, 0.1 mM CaCl_2_⋅2H_2_O) with 5% LB medium (Wang et al., 2017). *S. oneidensis* MR-1 cells in the mid-exponential growth phase were harvested by centrifugation, washed twice with M9 buffer, and re-suspended in the cathode electrolyte supplemented with 1.8 mM lactate. The prepared resting cells were inoculated to a final optical density at 600 nm (OD_600_) of 1.5, and fumarate in sufficient amount 40 mM was added as terminal electron acceptors. The cathode chamber was continuously purged with filtered nitrogen from the bottom of medium, and the MES system was maintained at room temperature with the constant stirring speed at 200 rpm. All the ES stock solutions were also pre-purged with filtered nitrogen for 2 h to remove dissolved oxygen before adding them to the MES system.

### Electrochemical Analysis

The amperometric i-t curve and cyclic voltammetry (CV) were monitored using a Chenhua CHI1030C potentiostat (Xafenias et al., 2016)*.* The potential of amperometric curve was imposed at −0.65 V (vs. Ag/AgCl). Unless otherwise specified, the CV assays were carried out in a conventional three-electrode system at a scan rate of 100 mV/s, in which the electrolyte was consisted of deoxygenated M9 buffer (pH 7.0) (Elgrishi et al., 2018). Current density and Coulombic efficiency in MES were calculated using the following equations (Steinbusch et al., 2010; Xafenias et al., 2016):

$${\text{Fumarate }+2\text{H}}^{+}+2e^{-}\to \text{Succinate}$$ (1)

Current density = I/S (2)

Coulombic efficiency=MnF/∫Idt×100% (3)

where I means current record, S means surface area of carbon cloth*,* M means the number of moles of products, n means the number of electrons in each reaction, and F is Faraday’s constant (96,485 C/mol of electrons). The production of succinate was analyzed by a high-performance liquid chromatography (HPLC) method on an Agilent 1,100 Series HPLC system equipped with a cation exchange column (Aminex HPX87-H, Bio-Rad, United States) as described previously (Bursac et al., 2017). Bioelectrochemical succinate production, referred to as the indicator of reduction product equivalent in MES, was determined by the increased yields of succinate in an energized condition compared to the unenergized condition. Results are representative of at least two independent experiments performed in triplicate and are expressed as mean ± standard deviation (SD).

### Quantum Chemical Calculations

The structures of substituted AQS derivatives and protonated water clusters in aqueous solution were explored using density functional theory (DFT) computations as described previously (Chen et al., 2013).

### Proton-Coupled Electron Transfer Reaction

**SCHEME 1:**
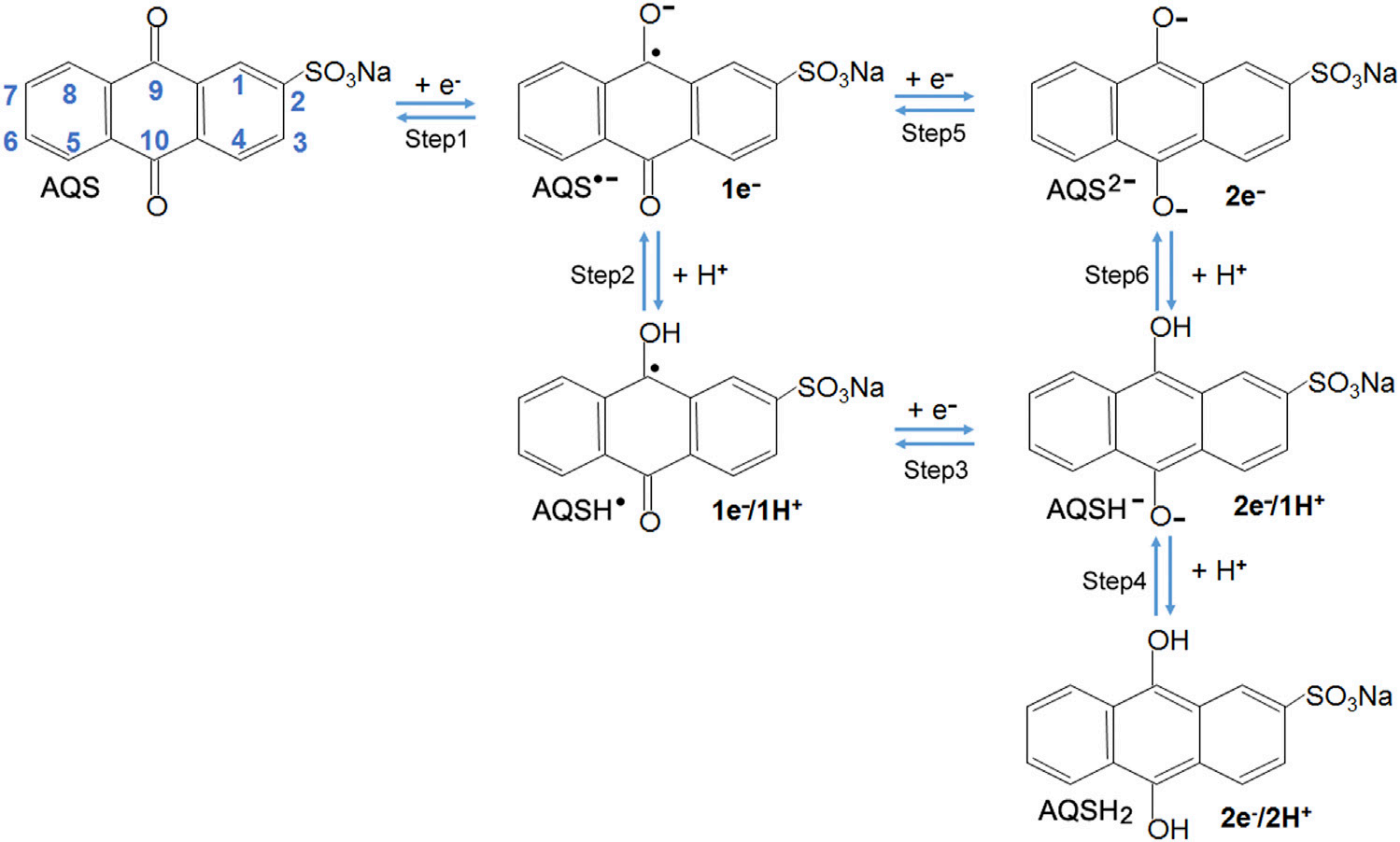
Possible steps of sequential electron and proton addition reactions for AQS in aqueous solution (Chen et al., 2013). Each AQS molecule accepts up to two electrons and is transformed into AQSH^−^ (2e^−^/1H^+^ process) or AQSH_2_ (2e^−^/2H^+^ process).

Due to the presence of redox-active moieties quinone groups, the redox reactions of AQS are referred to as proton-coupled electron transfer (PCET) (Migliore et al., 2014). As shown in Scheme 1, the possible steps of sequential electron and proton addition reactions for AQS in aqueous solution may occur via an incomplete 1e^−^/1H^+^ process or a complete 2e^−^/2H^+^ process, which basically is similar to the reduction process of phenazine-type ESs reported in the previous work (Chen et al., 2013). The detailed calculation procedures about Gibb’s free energy changes and redox potential were shown in Supplementary Material.

## Results and Discussion

### Evaluation of Different Electron Shuttles on Extracellular Electron Transfer Capacity of *S. oneidensis* MR-1

Despite the fact that high concentration of ESs might contribute to electron transfer, it can also result in toxicity effects (Rabaey and Rozendal, 2010). Here, a total 14 various ESs were selected to investigate their possible toxic effects on the growth of *S. oneidensis* MR-1. As shown in Table 1, different ESs showed the varying degree of influence on cell growth. At the concentration up to 0.05 mM, most ESs showed no apparent effects on bacterial cell growth. Five ESs including PMS, LV, NR, MB, and RF sharply decreased cell growth to below 75% of the untreated control. However, when the concentration of ESs reached 0.5 mM, 6 of 14 ESs reduced cell growth below 25% level, including NR, DCPIP, AR, LV, PMS, and MB. Notably, the addition of AQS, HA, and PH had no clear toxic effects on *S. oneidensis* MR-1 under the tested conditions.

**TABLE 1:** Effect of different ESs on cell growth of *S. oneidensis* MR-1.

Electron shuttles (ESs)^b^	Relative growth ability^a^				
	0.001 mM	0.01 mM	0.05 mM	0.1 mM	0.5 mM
NR	96.1 ± 1.7	86.5 ± 1.0	64.6 ± 0.5	53.8 ± 2.4	0
MV	102.2 ± 0.5	102.1 ± 2.7	94.8 ± 1.0	85.7 ± 1.4	36.7 ± 1.8
PH	101.3 ± 2.8	99.6 ± 2.1	97.7 ± 0.5	97.3 ± 3.9	91.8 ± 0.7
DCPIP	100.7 ± 0.4	101.4 ± 2.4	84.5 ± 2.9	63.5 ± 1.8	0
LV	65.6 ± 1.4	54.3 ± 4.2	21.3 ± 2.5	15.1 ± 6.3	0
TTC	95.3 ± 1.2	98.3 ± 3.1	96.1 ± 2.8	97.1 ± 1.0	83.6 ± 1.6
AR	100.0 ± 1.1	95.2 ± 4.5	92.2 ± 1.1	85.6 ± 2.6	15.4 ± 3.8
RF	93.8 ± 1.4	82.9 ± 4.2	71.5 ± 4.0	60.0 ± 2.8	45.2 ± 3.7
HA	100.8 ± 1.1	98.6 ± 4.2	100.1 ± 3.1	99.7 ± 3.0	96.9 ± 4.5
BV	101.9 ± 1.6	98.7 ± 4.9	95.1 ± 4.6	84.2 ± 2.7	38.4 ± 1.6
DTBBQ	94.1 ± 3.8	95.4 ± 1.8	92.1 ± 2.2	94.2 ± 2.2	66.6 ± 1.4
AQS	96.7 ± 4.1	105.5 ± 0.5	98.9 ± 0.6	102.2 ± 0.3	99.3 ± 1.8
PMS	102.6 ± 4.8	75.6 ± 2.4	3.8 ± 0.2	2.2 ± 0.2	0
MB	90.6 ± 2.5	87.9 ± 2.8	55.6 ± 5.9	55.7 ± 8.5	7.6 ± 0.3

a The relative growth ability was shown as percentage of cells at different concentrations of ESs compared to the untreated control, and the growth ability of *S. oneidensis* MR-1 cells without ESs was defined as 100%.

b Neutral red (NR), methyl viologen (MV), potassium hexacyanoferrate (PH), 2,6-dichlorophenolindophenol sodium salt hydrate (DCPIP), thionin acetate salt (LV), 2,3,5-triphenyltetrazoliun chloride (TTC), alizarin red (AR), riboflavin (RF), humic acid (HA), benzyl viologen dichloride (BV), 2,6-di-tert-butyl-1,4-benzo-quinone (DTBBQ), anthraquinone-2-sulfonate (AQS), phenazine methosulfate (PMS), methylene blue (MB).

In order to investigate the effects of these ESs on electron transfer efficiency, the H-shaped double chamber MES system was constructed with *S. oneidensis* MR-1 as the microbial catalyst and fumarate as the terminal electron acceptor. For taking into account the toxicity effects of ESs, 0.05 mMESs was used in the follow-up study. As shown in Supplementary Figure S1B, there was a smooth background current less than 10 μA when the cathodic potential was imposed at −0.65 V (vs. Ag/AgCl [3M KCl]). The presence of fumarate slightly increased the current to 50 μA, supporting that *S. oneidensis* MR-1 can directly uptake electrons from a cathode. A more significant current response was observed when 50 μM of a well-characterized electron shuttle RF was added. The highest current was up to 7 mA and was 140 times higher than that without the addition of RF. Moreover, the CV assays in the MES reactor showed that the current was remarkably enhanced with the increase of the cathodic potential in the presence of RF (Supplementary Figure S1C).

**FIGURE 1:**
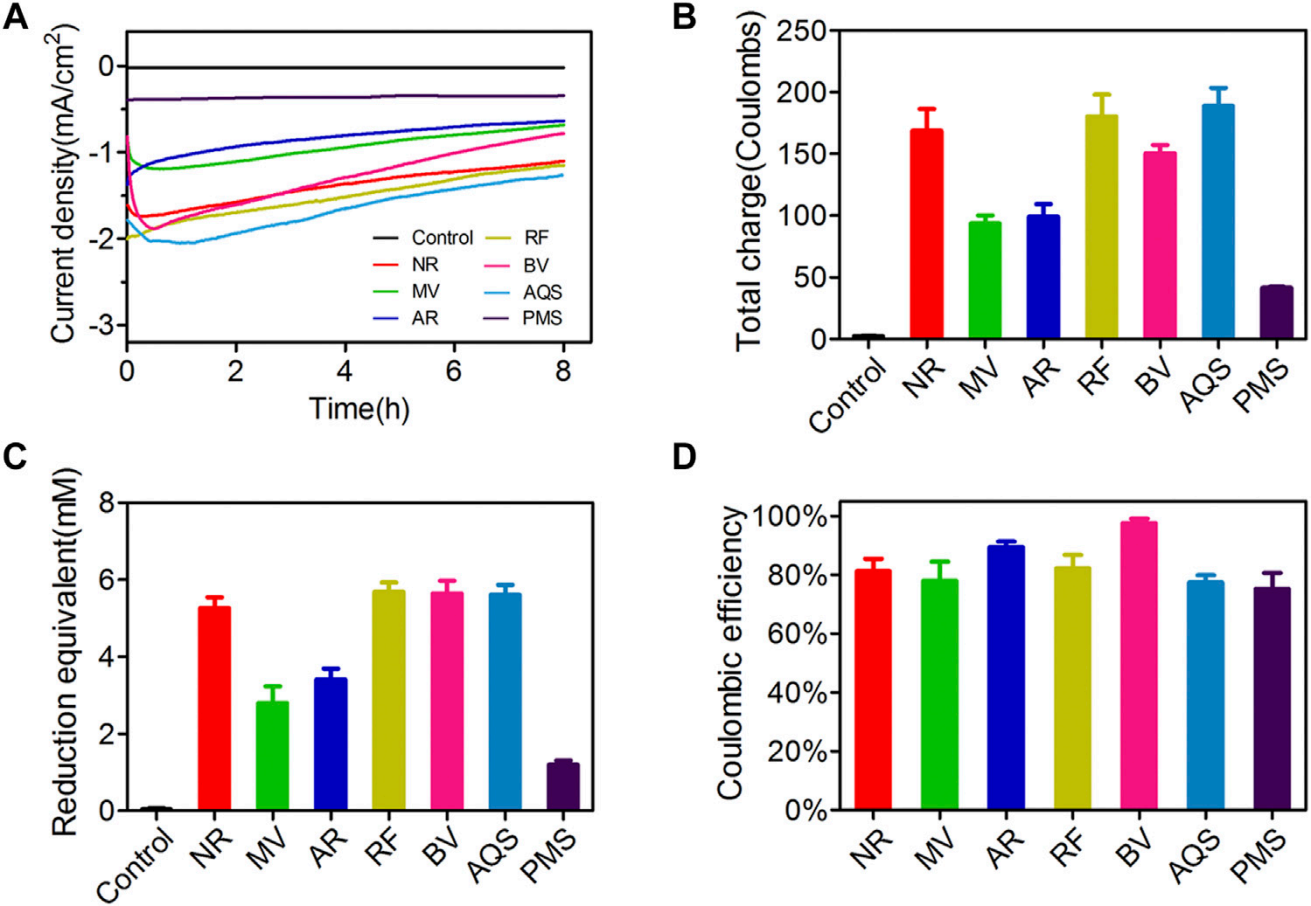
The electrochemical properties of different ESs in MES. **(A)** Current produced by a cathode with or without ESs addition was recorded by the CHI1030C potentiostat under the potential of −0.65 V (vs. Ag/AgCl). Current density was calculated using the cathodic surface area of carbon cloth. **(B)** Total charge production was determined in the end of 8-h electrosynthesis period. **(C)** The production of succinate was used as an important indicator of reduction product equivalent, which was examined by the HPLC method. **(D)** Coulombic efficiency was calculated according to the equation in “Materials and Methods” section. Results are representative of at least two independent experiments performed in triplicate and are expressed as mean ± standard deviation (SD).

Half of ESs were found to have obvious current responses in a range from 1.6 to 8 mA, including NR, MV, AR, RF, BV, AQS, and PMS. To better examine the electrochemical properties of different ESs in the enhancement of cathodic electron uptake, we performed an 8-h electrosynthesis period during which microbial biomass was not noticeably affected by electricity. As shown in Figure 1A, all these above ESs had a current density over 0.4 mA/cm^2^, while the current density of the control without the addition of ESs was only about 0.02 mA/cm^2^. In comparison, the other half of ESs showed the highest current density less than 0.06 mA/cm^2^ **(** Supplementary Figure S2). As shown in Figure 1B, the produced total charges of tested ESs were 168.6 ± 17.8 C for NR, 93.6 ± 6.5 C for MV, 99.2 ± 10.1 C for AR, 180.4 ± 17.5 C for RF, 150.6 ± 6.5 C for BV, 188.9 ± 14.6 C for AQS and 41.6 ± 0.8 C for PMS, respectively, which were much higher than that of the control without ESs. These results suggested that these ESs could effectively increase the ability of current response and continuously promote charge accumulation in MES. The reduction product equivalents, referred to as the altered production of succinate after the impulse of electrode potential, were also examined (Figure 1C). All tested ESs except PMS sharply increased the concentration of reduction equivalents in MES to above 2.5 mM, whereas the control without ESs had almost no capacity to produce succinate (less than 0.05 mM). The Coulombic efficiencies mediated by ESs were also calculated as the percentage of supplied electrons that were converted to succinate product (Figure 1D). The Coulombic efficiencies in the presence of these ESs were over 75%, implying that the addition of ESs to a cathode contributed to improving the efficiency of succinate production. Notably, the control MES system without ESs showed an illogical Coulombic efficiency of 127.5 ± 30.4%, which means that the number of moles of electrons in the conversion of succinate is greater than the measured charges derived from the cathodic current data. This phenomenon has also been mentioned in several other studies (Liu et al., 2013; Harrington et al., 2015a). A plausible explanation was that the number of electrons from cathode was not the only source for the increased succinate in MES. The electricity-driven reducing power might also affect intracellular redox homeostasis and metabolism, thereby leading to an unexpected increase of reduced metabolites products such as succinate. Given that the control system exhibited the current density and succinate accumulation at quite low levels, the altered production of succinate caused by cellular metabolic disorder with electricity would inevitably interfere with the correct calculation of its Coulombic efficiency.

### Determination of the Redox Potentials of Different Electron Shuttles

The redox potential of ESs is an important electric indicator to affect electron transfer between the electrode and the solution redox species (Van der Zee and Cervantes, 2009; Wu et al., 2014). In order to better understand the property of distinct ESs, the redox potentials of seven ESs were measured by the CV assays under experimental conditions (Table 2; Supplementary Figure S3). The results showed that all ESs had reversible redox peaks, and the redox potentials were below the boundary potential level of fumarate reduction reaction (−0.227 V). As shown in Table 2, PMS had a near boundary value of redox potential (−0.24 V) and showed a Coulombic efficiency of 75.6%. AQS had a highly negative redox potential (−0.46 V) with its Coulombic efficiency rising to 77.4%. Moreover, NR and AR exhibited more negative redox potential, with higher Coulombic efficiency of up to 81.3 and 89.5%. The redox potential of BV was −0.58 V, and the Coulombic efficiency of BV could reach values of over 97% in MES system. These data supported a possibility that the ES with a more negative potential in a certain range may be conducive to the improvement of Coulombic efficiency in MES.

**TABLE 2:** Redox potentials of ESs and Coulombic efficiencies in MES.

Electron shuttles	Standard redox potential^a^(V vs. Ag/AgCl)	Experimental redox potential (V vs. Ag/AgCl)	Coulombic efficiency
NR	−0.54	−0.56 ± 0.04	81.3 ± 4.2%
MV	−0.65	−0.68 ± 0.08	78.0 ± 6.5%
AR	−0.55	−0.56 ± 0.03	89.5 ± 1.8%
RF	−0.42	−0.44 ± 0.02	82.2 ± 4.6%
BV	−0.57	−0.58 ± 0.06	97.6 ± 1.5%
AQS	−0.44	−0.46 ± 0.02	77.4 ± 2.4%
PMS	—	−0.24 ± 0.07	75.6 ± 5.4%

a The standard redox potential was derived from the previous studies (The references were listed in the Supplementary Matreial), and the electrode potential of Ag/AgCl (3M KCl) against standard hydrogen electrode (SHE) is +0.210 V.

### Design of Anthraquinone-2-Sulfonate With Functional Groups to Change the Redox Potentials

Based on the above evaluations on the properties of ESs (Figure 1; Table 1), AQS was revealed no obvious toxic effects on *S. oneidensis* MR-1, and led to the highest current density (2.04 mA/cm^2^) and total charges (188.9 ± 14.6 Coulombs). These properties supported a fact that AQS was one of the most prosing electron shuttles for cathodic EET process in *S. oneidensis* MR-1. However, the Coulombic efficiency in MES with AQS addition was still relatively low. Thus, the manipulation of redox potential of AQS by changing molecular structure might provide a new strategy to reduce energy losses and increase the electron-product conversion efficiency in MES.

**FIGURE 2:**
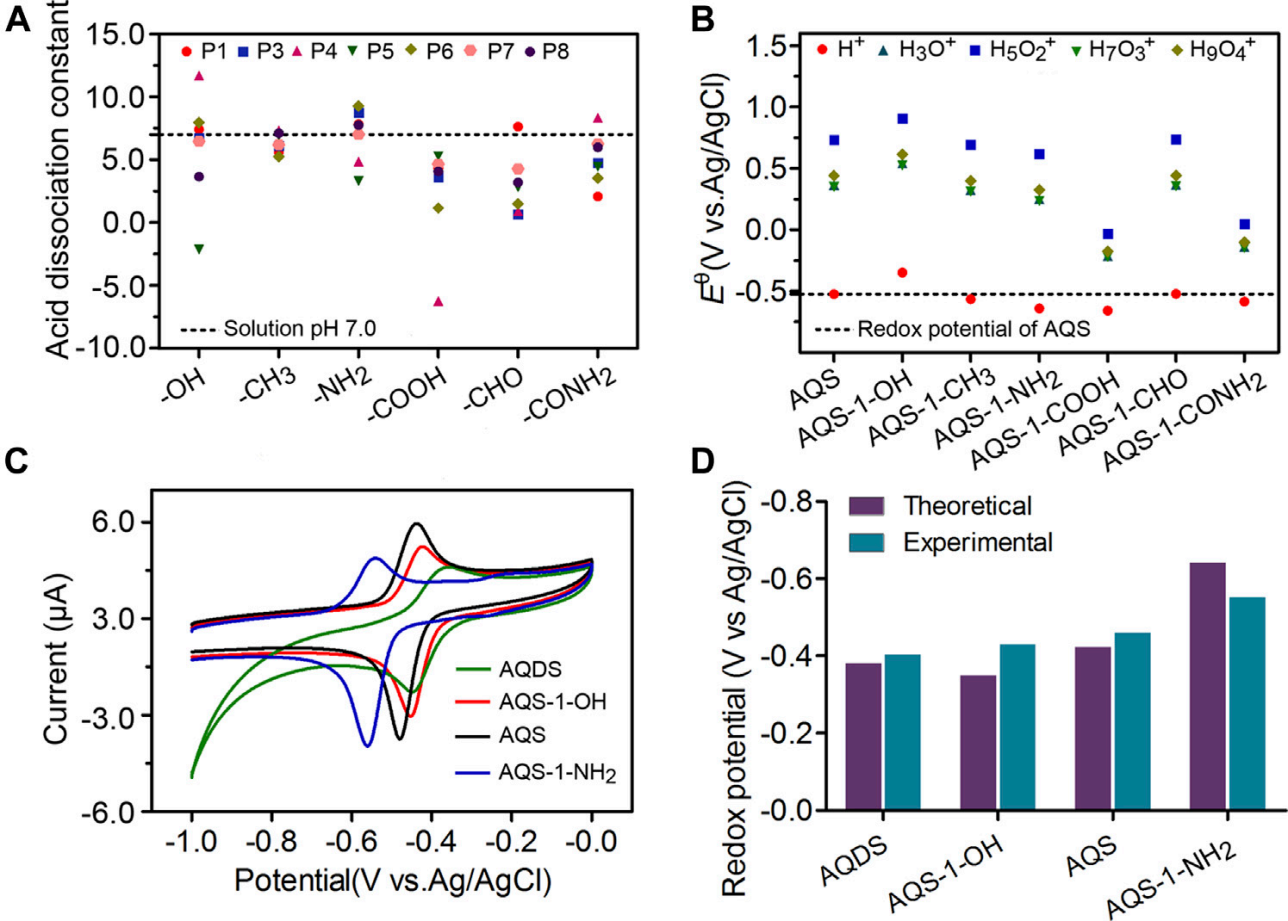
Effects of different substituents on the redox potentials of AQS derivatives. **(A)** The acid dissociation constant pKa of AQSH_2_ species in the presence of different nature and position of the substituent groups. The P1 to P8 indicates the position of functional groups. **(B)** The redox potentials in aqueous solution of different AQS-1 derivatives. **(C)** The CV assays of AQS and three other derivatives. **(D)** The theoretical redox potentials were determined by the quantum chemical calculation, and the experimental redox potentials were calculated based on the CV assay. Results are representative of at least two independent experiments performed in triplicate and are expressed as mean ± standard deviation (SD).

Each AQS molecule can accept up to two electrons and be transformed into AQSH^−^ (2e^−^/1H^+^ process) or AQSH_2_ (2e^−^/2H^+^[3M KCl] process) by the PCET reaction. The presence of protonated water clusters, including H_3_O^+^, H_5_O_2_^+^, H_7_O_3_^+^, H_9_O_4_^+^, may affect the PCET reaction, and also considered in the quantum-chemical calculations. The detailed Gibb’s free energy changes of AQSH^−^ and AQSH_2_ species in aqueous solution were shown in Supplementary Tables S3, S4 in Supporting Information, respectively. In the water system, the formation of AQSH^−^ and AQSH_2_ species typically depends on the solution pH and the pKa of the neutral AQSH_2_. If the solution pH is lower than the pKa, the molecule is mostly deprotonated, and if the solution pH is higher than the pKa, the molecule is mostly protonated. According to the Henderson-Hasselbalch equation, the pKa of AQS derivatives with different substituents was calculated. As shown in Figure 2A, the majority of AQS substituents showed pKa values below the solution pH of 7.0, indicating that most molecules can exist in the form of AQSH^−^ (2e^−^/1H^+^) under the electrochemical conditions.

Given that the AQS molecule contains sulfonate group at 2-position, there are seven different monosubstituted positions in the molecular structure, including 1-, 3-, 4-, 5-, 6-, 7-, and 8-position (Scheme 1). In this study, quantum-chemical calculations based on the DFT computations were performed to explore the impacts of the substituent group on the redox potentials, and six different functional groups (-CH_3_, -CHO, -CONH_2_, -COOH, -NH_2_, and -OH) at each position were evaluated. According to the Nernst equation, the redox potentials of AQS derivatives were calculated from *Δ*G (2e^−^/1H^+^). Compared with the standard redox potential of AQS, three substituent groups (–OH, –NH_2_, and–COOH) led to significant changes on the redox potentials of AQS derivatives at the positions 1, 4, 5, and 8 close to the reactive quinone groups (Figure 2B and Supplementary Figure S4). In consideration of the changes of the redox potentials and the difficulties of chemical synthesis, we obtained AQS-1-OH and AQS-1-NH_2_ by a chemical method (Supplementary Figure S5). In addition, the structural analogous AQDS was also selected for further measurements. The redox potentials of these chemicals in the electrochemical experiments were examined by the CV assays (Figure 2C). Consistent with the theoretical calculations, the measured results showed that AQS-1-OH had a higher redox potential (−0.44 V) than that of AQS (−0.46 V), whereas AQS-1-NH_2_ had a lower redox potential (−0.55 V) (Figure 2D). Moreover, the tested redox potential of AQDS showed less negative than that of AQS, which was also in accordance with the reported redox potentials. Taken together, these data confirmed that the addition of certain functional groups significantly affected the redox potential of ESs.

### Effects of Anthraquinone-2-Sulfonate Derivatives on the Coulombic Efficiency

**FIGURE 3:**
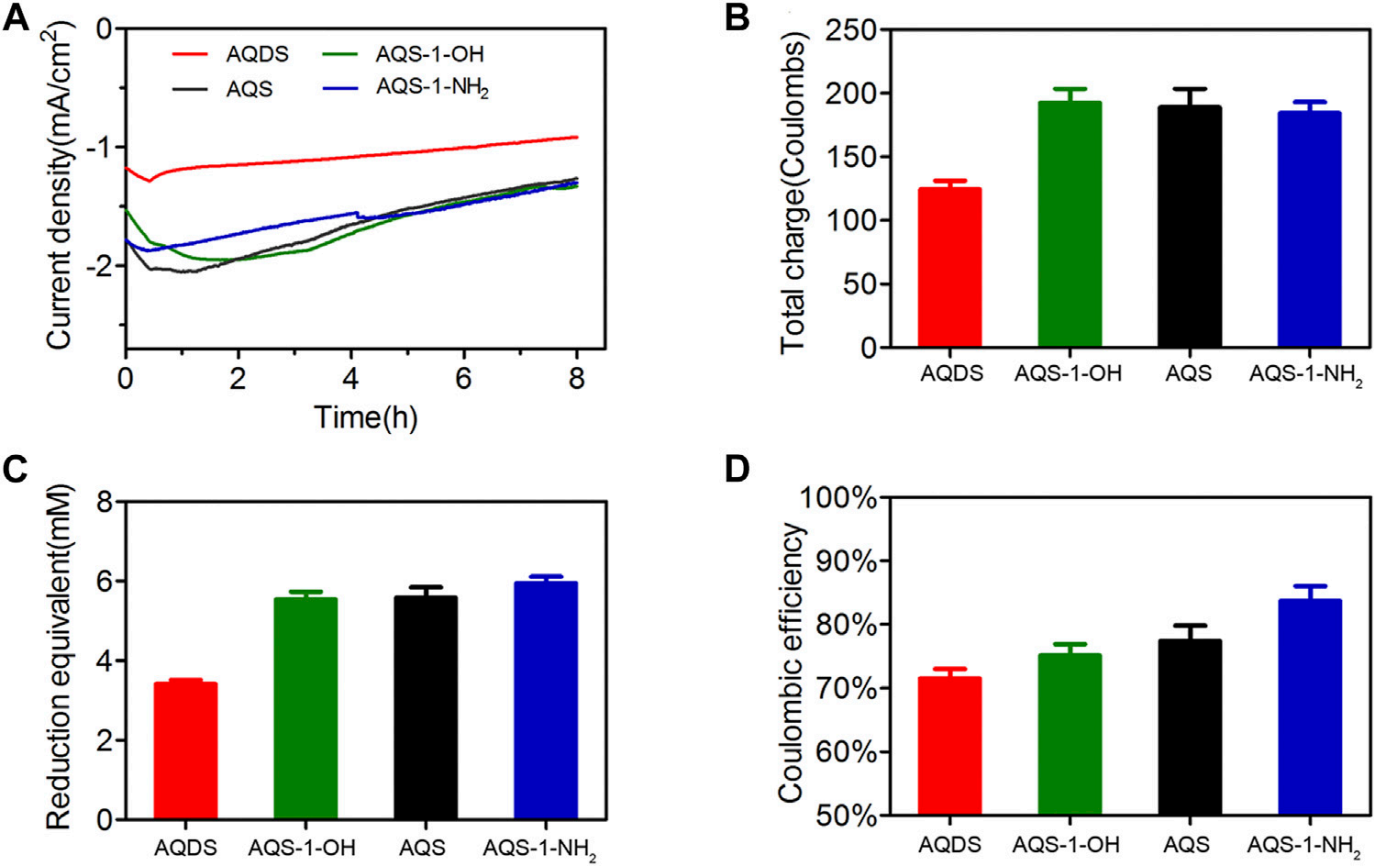
The electrochemical behaviors of AQS and derivatives in MES. **(A)** Current density produced by a cathode in the presence of different AQS derivatives under the potential of −0.65 V (vs. Ag/AgCl). **(B)** Total charge production produced by a cathode after 8-h electrosynthesis period. **(C)** Reduction product equivalent (succinate production) produced in MES after 8-h electrosynthesis period. **(D)** Coulombic efficiency of MES in the presence of different AQS derivatives. Results are representative of at least two independent experiments performed in triplicate and are expressed as mean ± standard deviation (SD).

To test the possibility that the designed ES with a more negative redox potential has a low energy loss and high conversion efficiency, the effects of AQS and three other derivatives on the cathodic EET process were evaluated in MES. In cell toxicity tests, these four ESs showed limited inhibitory effects on *S. oneidensis* MR-1 growth at the concentration of ESs below 0.1 mM, and only AQDS had a little cell toxicity when the AQDS concentration was over 0.5 mM (Supplementary Figure S6). Current response could also be observed when these four ESs were added into the MES, indicating that these ESs had the ability to transfer electrons from a cathode to the fumarate acceptor. As shown in Figure 3A, the current densities of AQS-1-OH and AQS-1-NH_2_ during an 8-h electrochemical reduction process were almost equally with that of AQS, while AQDS had the minimum current density. Consistent with the current density, the total charges produced by AQDS, AQS-1-OH, AQS, and AQS-1-NH_2_ within 8 h were 124.3 ± 6.8, 192.3 ± 11.2, 188.9 ± 14.6, and 184.2 ± 8.9°C, respectively (Figure 3B). Almost equal reduction product equivalents were produced in MES reactors with AQS-1-OH, AQS, and AQS-1-NH_2_, which were approximately 62% higher than that of AQDS (Figure 3C). Based on the data of total charges and reduction product equivalents, the Coulombic efficiencies in the presence of AQDS, AQS-1-OH, AQS, or AQS-1-NH_2_ was 71.5 ± 1.5, 75.1 ± 1.8, 77.4 ± 2.4, and 83.7 ± 2.3%, respectively (Figure 3D).

The EET process from the reduced ESs to the electron acceptors of *S. oneidensis* MR-1 is considered as a rate-limiting step, which might be attributed to the hypothesis that reduced ESs should directly contact cytochromes on the outer membrane or penetrate through cell membranes for efficient electron transfer (Baron et al., 2009; Zhao et al., 2020). According to the principle of redox reaction, the enlargement of redox potential difference between the reduced ESs and the electron acceptor will be helpful for a complete reaction at sufficient rates (Chen et al., 2013). Consistent with these theories, the AQS-1-NH_2_ showed a lower redox potential and a higher Coulombic efficiency than those of AQS derivatives, supporting that a more negative redox potential of ES derivatives by substituents contributes to the high reaction efficiency.

### Essential Roles of the Mtr Pathway on the Anthraquinone-2-Sulfonate-Mediated Extracellular Electron Transfer Process

The previous work reveals that MV cannot penetrate the cytoplasmic membrane of microorganisms, and can only participate electrochemical interactions with the periplasm (Rosenbaum et al., 2011). Unlike MV, NR is shown to bind to cell membranes and subsequently be oxidized by fumarate reductase or intracellular NAD^+^ (Park and Zeikus, 1999; Harrington et al., 2015b). Tokunou et al. (2016) found that two flavin-like polycyclic compounds bearing a shorter side chain than riboflavin, safranin and anthraquinone-1-sulfonate, could increase cathodic current production by functioning as bound redox cofactors in the outer membrane c-type cytochrome OmcA. However, the underlying mechanisms of other ESs-mediated EET still need to be explored further. Many studies have reported that the Mtr pathway is required for the direct electron transfer to or from electrodes (Kracke et al., 2015; Rowe et al., 2018; Gong et al., 2020). At least five primary protein components have been identified for the Mtr pathway in *S. oneidensis*, including four c-type cytochromes OmcA, MtrC, MtrA and CymA, and one integral scaffolding protein, MtrB (Yang et al., 2012). Although recent studies showed that the Mtr pathway played an important role in direct EET process from a cathode to *S. oneidensis* MR-1, little is known about the AQS-mediated EET process in MES. Especially in such a case that the addition of AQS into MES increased cathodic EET capacity by at least 100 times than that of the control without ESs (Figure 1A). Therefore, a better understanding of the AQS-mediated EET mechanism might provide new insights into bacterial electron transfer.

**FIGURE 4:**
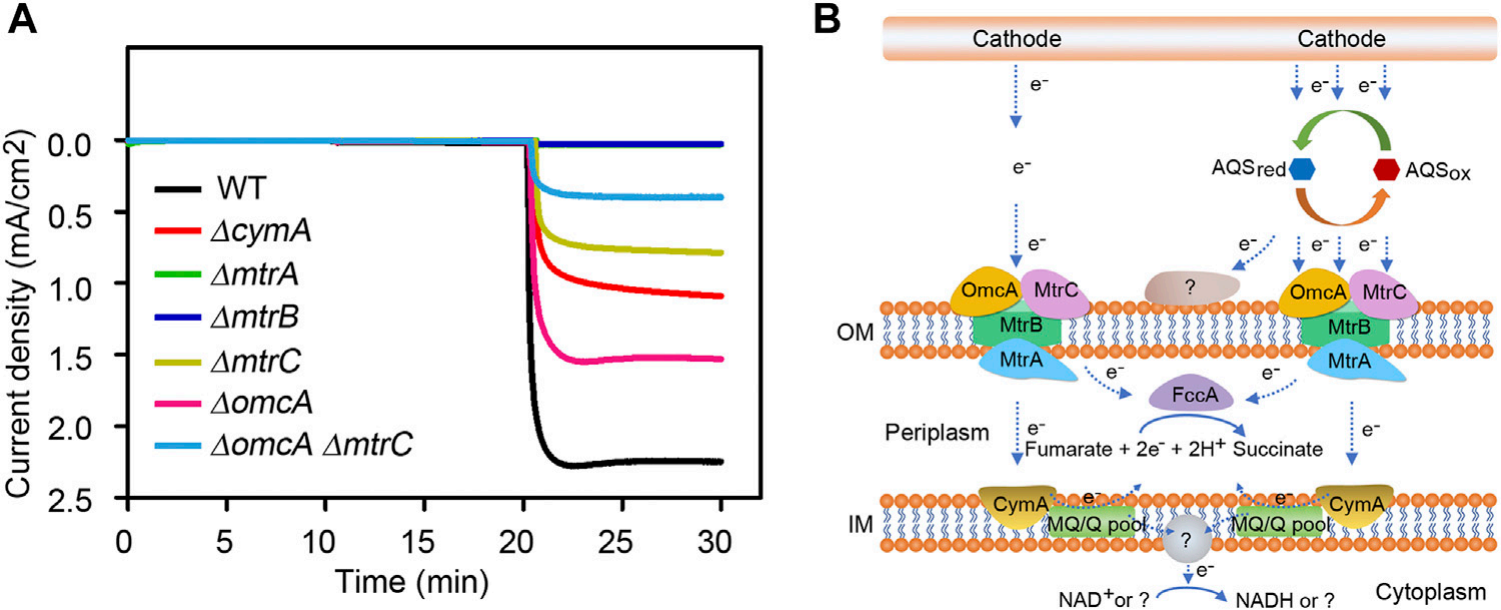
Role of Mtr pathway in AQS-mediated cathodic EET process of *S. oneidensis* MR-1. **(A)** Measurements of current density in deletion mutants lacking different Mtr-associated proteins. **(B)** A simple model on the AQS-mediated cathodic EET mechanism? symbol indicates unidentified enzyme, substrate or product.

In order to explore the possible roles of Mtr pathway on the AQS-mediated EET process, six deletion mutants were constructed by an in-frame mutagenesis strategy, including the single mutants of *∆omcA*, *∆mtrA*, *∆mtrB*, *∆mtrC*, *∆cymA* and the double mutant of *∆omcA ∆mtrC*. As shown in Figure 4A and Supplementary Figure S7, all the single-gene deletion mutants displayed significant decreases in the cathodic current density compared to wild-type strain, and the mutant phenotypes could be reverted to approximately 70–90% of wild-type levels by introducing the corresponding endogenous gene under the control of the IPTG-inducible tac promoter. Among them, the current density of the *∆mtrA* or *∆mtrB* mutant was greatly decreased by approximately 99%, indicating that the cathodic EET capacity mediated by AQS has completely been lost when either MtrA or MtrB protein is absent. Moreover, the single deletion of *mtrC* or *omcA* resulted in approximately 63% or 27% current drop, whereas the *∆omcA ∆mtrC* double mutant showed more than ∼82% reduction in cathodic current density as compared with the wild-type strain. This finding suggested that OmcA and MtrC played a crucial role in the delivery of electrons to cells, but the function of other unidentified membrane proteins were also needed to be investigated. In addition, loss of the inner membrane-associated CymA also led to 48% decrease in the current density. In conclusion, we proposed a simple model about the AQS-mediated EET pathway (Figure 4B). In *S. oneidensis* MR-1, the Mtr respiratory pathway is proved to be involved in the direct electron transfer from electrodes, but electron shuttles provide great promise for obtaining the outstanding EET capacity. In the case of AQS, the oxidized form AQSox can be converted to its reduced form AQSred by accepting two electrons from the electrode. The reduced form of AQS is re-oxidized by transferring electrons to the outer membrane MtrC-OmcA cytochromes and other as-yet-unidentified components. Almost all the released electrons are then transferred to the periplasmic space through the components MtrB and MtrA. As the sole fumarate reductase located in the periplasm, FccA allows the catalysis of fumarate as a terminal electron acceptor to generate succinate in anaerobic environment. CymA has been considered as an inner membrane electron-distribution hub, exhibiting the capacity to deliver electrons back to periplasmic FccA via the quinone pool in the inner membrane (Kracke et al., 2015). Besides, a portion of reverse electron flow derived from the cathode may also have chance of ultimately passing through plasma membrane to generate the reducing equivalents. Taken together, we found that the MtrA and MtrB components were essential for the AQS-mediated EET of *S. oneidensis* MR-1, and the c-type cytochromes OmcA and MtrC accounted for partially 82% of cathodic electron flows. These findings revealed that the AQS-mediated electrons was majorly transported into the periplasmic space by the outer-membrane-bound OmcA-MtrCAB complex. Although more components will still need to be fully characterized in future research, our report provides a preliminary understanding of the AQS-mediated EET process of *S. oneidensis* MR-1.

In summary, here we want to provide specific clues for the bioengineering and improvement of electron shuttles and Mtr respiratory pathway, aiming to increase electron transfer capability from the electrode towards microbes. These attempts were believed to move the field of ESs design into synthetic biology, especially for the modification of the synthetic pathways of endogenous electron shuttles. For example, if quantum-chemical prediction of redox potentials reveals that the introduction of specific substituent groups into endogenous ESs (such as flavin or phenazine) facilitates effective electron transfer between microbes and the electrode, this may offer us inspiration to redesign and construct novel artificial biological pathways for an ideal ESs derivative using synthetic biology strategies. Additionally, the findings also indicated that the Mtr pathway was a rate-limiting step for AQS-mediated EET process, providing enabling insights into the engineering of electroactive microorganisms for better bioelectrical properties.

## Conclusion

Electron shuttles play important roles in promoting the EET efficiency between the electrode and microorganisms. Given that the electrochemical properties of various ESs might be distinct in MES, the screen of optimal ESs for specific electro-active bacteria will contribute to the highly efficient electron transfer to end-product production. In this work, we found that AQS might function as the optimal ES for enhancing the cathodic EET capacity of *S. oneidensis* MR-1 with the highest current density, total charge production and reduction product equivalent compared with other tested ESs. Altering the redox potential of AQS to more negative by the addition of specific substituent groups to polycyclic backbone is propitious to the reduction half-reaction of electron acceptor at sufficient rates, providing a new alternative strategy for the acceleration of EET process in *S. oneidensis* MR-1. Further studies suggested that the Mtr respiratory pathway, especially the MtrA and MtrB components, is essential for the AQS-mediated EET in *S. oneidensis* MR-1.

## Data Availability

The raw data supporting the conclusion of this article will be made available by the authors, without undue reservation.
