# Supplementary material for: Tuning Redox Potential of Anthraquinone-2-Sulfonate (AQS) by Chemical Modification to Facilitate Electron Transfer From Electrodes in Shewanella oneidensis
Source: Front Bioeng Biotechnol. 2021 Aug 10;9:705414. doi: 10.3389/fbioe.2021.705414 (PMC8383453; doi:10.3389/fbioe.2021.705414)
Supplement: Supplementary file 1 [file DataSheet1.docx]

**Supporting Information**

**Tuning redox potential of anthraquinone-2-sulfonate (AQS) by chemical modification to facilitate electron transfer from electrodes in *Shewanella oneidensis***

**Ning Xu ^1, 4 †^, Tai-Lin Wang ^1, 3†^, Wen-Jie Li ^2 †^, Yan Wang ^1, 3^, Jie-Jie Chen ^2 *^, Jun Liu ^1, 3, 4*^**

^1^ Tianjin Institute of Industrial Biotechnology, Chinese Academy of Sciences, Tianjin 300308, P. R. China

^2^ Key Laboratory of Urban Pollutant Conversion, Department of Applied Chemistry, University of Science and Technology of China, Hefei 230026, P. R. China

^3^ University of Chinese Academy of Sciences, Beijing 100049, P. R. China.

^4^ Key Laboratory of Systems Microbial Biotechnology, Chinese Academy of Sciences, Tianjin 300308, P. R. China.

†These authors have contributed equally to this work.

*** Correspondence:**

Jun Liu, E-mail: liu_jun@tib.cas.cn

Jie-Jie Chen, E-mail: chenjiej@ustc.edu.cn

**Supplementary information includes:**

7 figures; 4 tables

**Materials and Methods:**

According to the reaction steps, the Gibb’s free energy changes of an incomplete 1e^-^/1H^+^ process in aqueous solution can be obtained by summarizing step 1 and step 2:

Δ*G* (1e^-^/1H^+^) = Δ*G*_1_ +Δ*G*_2_ (1)

The Gibb’s free energy changes of a complete 2e^-^/2H^+^ process can be calculated from 4 steps by the following equations:

*ΔG* (2e^-^/2H^+^) = *ΔG*_1_ +*ΔG*_2_ +*ΔG*_3_ +*ΔG*_4_ (or = *ΔG*_1_ +*ΔG*_5_ +*ΔG*_6_ +*ΔG*_4_) (2)

The addition of a second proton depends on the solution pH and the pKa of the neutral AQSH_2_, and the Gibb’s free energy changes of a 2e^-^/1H^+^ process was calculated by the following equations:

Δ*G* (2e^-^/2H^+^) = Δ*G*_1_ +Δ*G*_2_ +Δ*G*_3_ (or = Δ*G*_1_ +Δ*G*_5_ +Δ*G*_6_) (3)

The p*K*a values of the protonated species can be calculated from the Δ*G* of proton additional steps by the following Henderson-Hasselbalch equation:

p*K*a = -Δ*G*/2.303*RT* (4)

The redox potentials in aqueous solutions for 2e^-^/1H^+^ or 2e^-^/2H^+^ reductions of AQS derivatives were calculated by the following Nernst equation:

*E* = -Δ*G*/*nF* – *E*_H_ (5)

where *n* is the number of electrons from oxidative AQS derivatives to reductive AQS derivatives, *F* is the Faraday constant with a value of 23.06 kcal/mol V, and *E*_H_ is the standard reduction potential of normal hydrogen electrode with a vaulue of 4.28 V.

All electrochemical experiments of ESs were conducted in M9 buffer (pH = 7.0) at room temperature and atmospheric, which is consistent to the calculation settings under standard condition.

**References for standard redox potential of ESs in Table 2**

Li, S., Song, Y.E., Baek, J., Im, H.S., Sakuntala, M., Kim, M., et al. 2020. Bioelectrosynthetic conversion of CO_2_ using different redox mediators: electron and carbon balances in a bioelectrochemical system. *Energies*, 13, 2572. doi: 10.3390/en13102572

Wolf, M., Kappler, A., Jiang, J., Meckenstock, R.U. 2009. Effects of humic substances and quinones at low concentrations on ferrihydrite reduction by Geobacter metallireducens. *Environ Sci Technol*, 2009, 43: 5679-85. doi: 10.1021/es803647r.

Marsili, E., Baron, D.B., Shikhare, I.D., Coursolle, D., Gralnick, J.A., Bond, D.R. 2008. *Shewanella* secretes flavins that mediate extracellular electron transfer. *Proc Natl Acad Sci U S A*, 105: 3968-73. doi: 10.1073/pnas.0710525105.

Badalyan, A., Yang, Z.-Y., Seefeldt, L.C. 2019. A Voltammetric Study of Nitrogenase Catalysis Using Electron Transfer Mediators. *ACS Catalysis*, 9, 1366-1372. doi: 10.1021/acscatal.8b04290

Tang, X.H., Ng, H.Y. 2017. Anthraquinone-2-sulfonate immobilized to conductive polypyrrole hydrogel as a bioanode to enhance power production in microbial fuel cell. *Bioresour Technol*, 244, 452-455. 10.1016/j.biortech.2017.07.189

**Supplementary figures:**


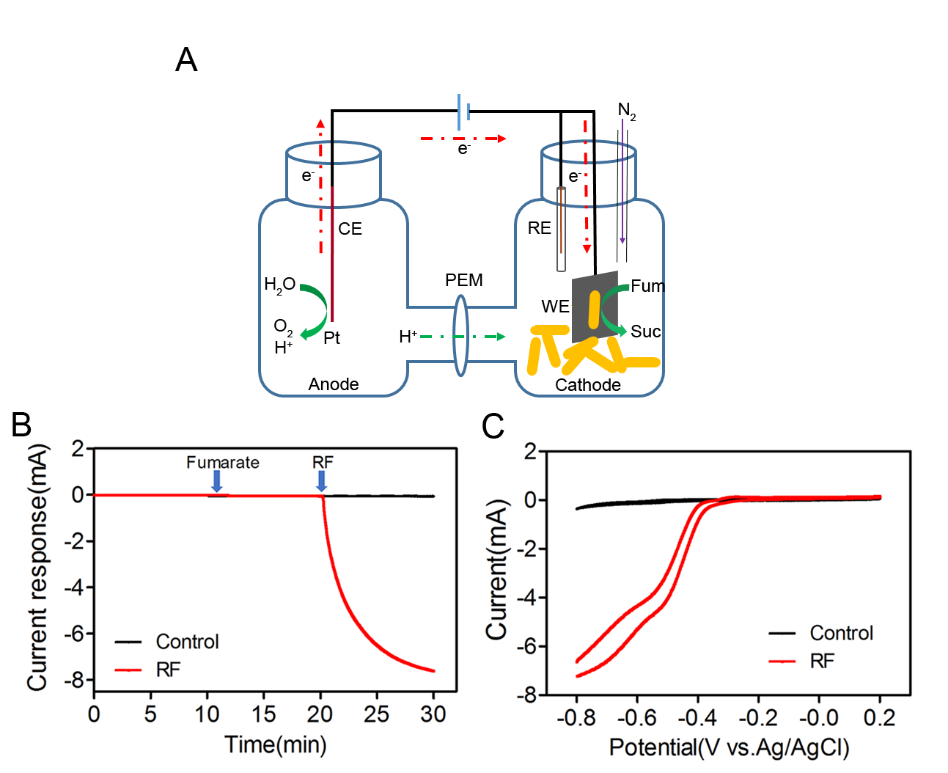


**Figure S1**. The construction and operation of double-chamber MESs with a working volume of 135 mL in each side. **(A)** MES reaction setup. A Pt counter electrode was placed in an anode chamber. A 4-cm^2^ carbon cloth and an Ag/AgCl reference electrode were placed in a cathode chamber. *S. oneidensis* MR-1 cells were suspended in the cathode electrolyte supplemented with 1.8 mM lactate. **(B)** Current response produced by a cathode under the potential of -0.65 V (vs Ag/AgCl) when 50 μM RF was added. 40 mM fumarate was purged with filtered nitrogen for 2 hours, and pre-added into the cathode chamber after 10-min operation of the reactor. The MES reactor without RF addition was used as a control. **(C)** Voltammetric studies of RF in the MES reactor with fumarate as the electron acceptor. The biotic reactor without RF addition was used as a control.


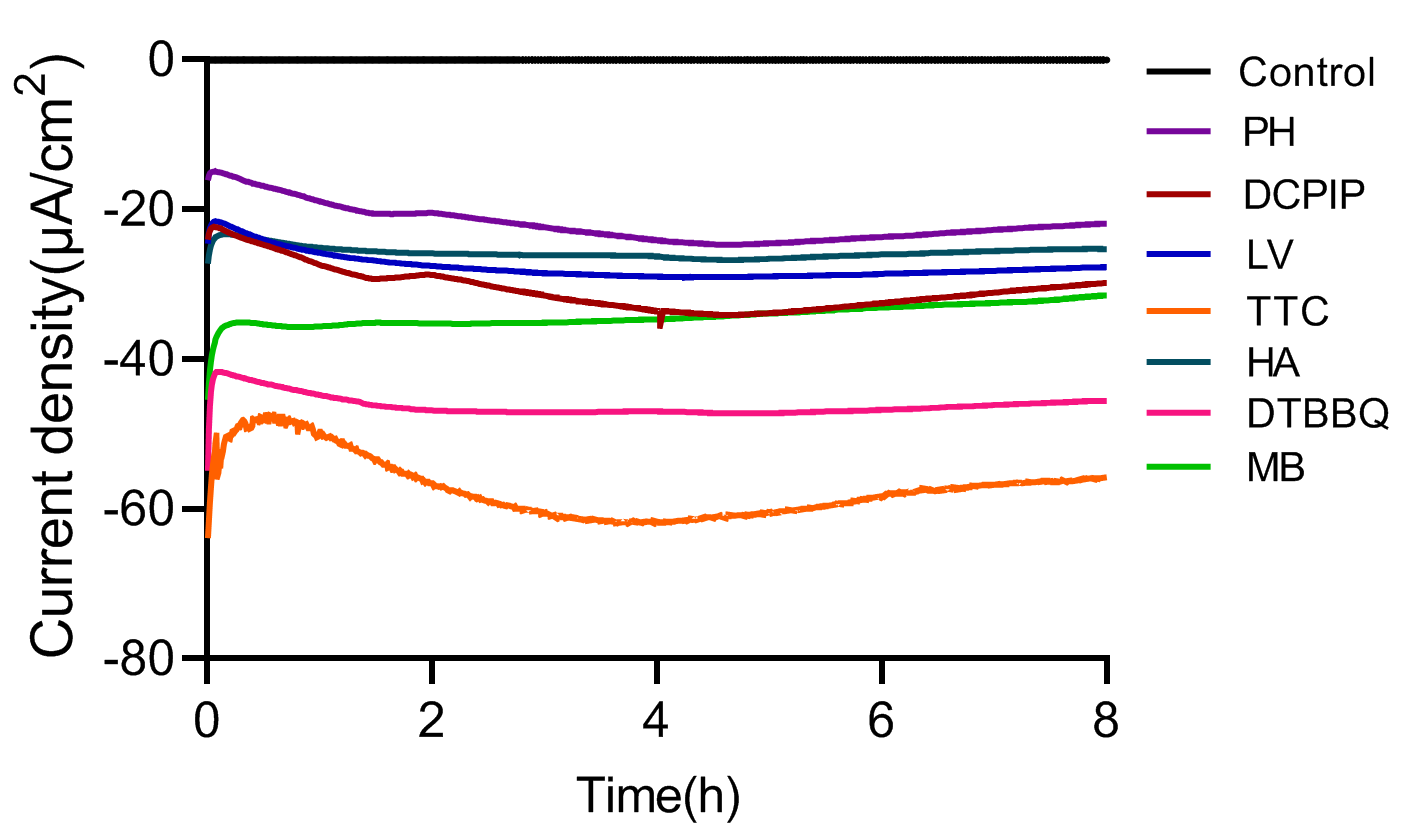


**Figure S2.** The electrochemical properties of the other seven ESs in MES. Current produced by a cathode with indicated ESs was recorded by the CHI1030C potentiostat under the potential of -0.65 V (vs Ag/AgCl). The current densities were calculated using the anodic surface area of 4 cm^2^ carbon cloth.


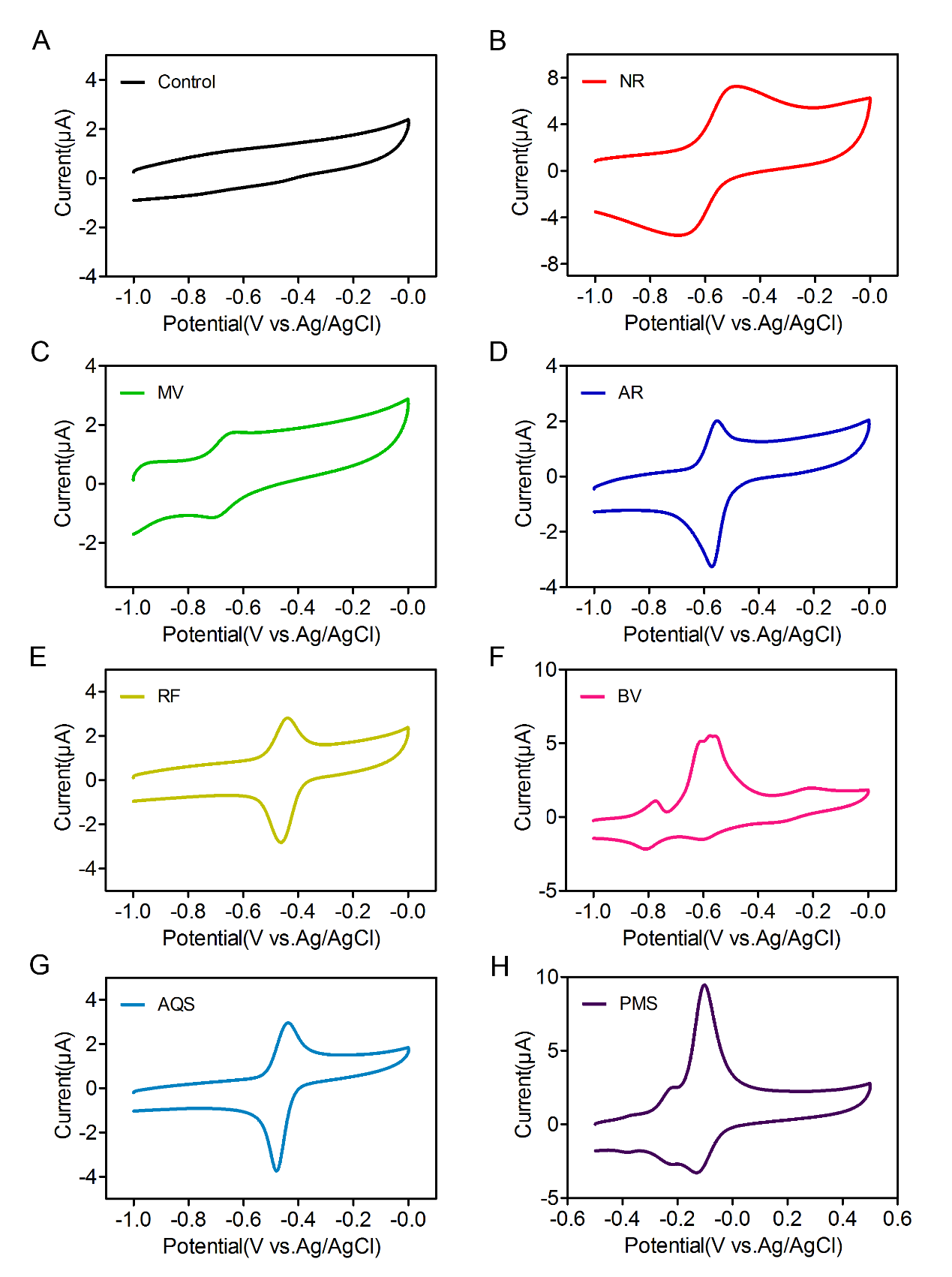


**Figure S3.** The CV assays of without and with different ESs. The assays were monitored by the CHI1030C potentiostat at a scan rate of 100 mV/s using a three-electrode arrangement in a single chamber that composed of deoxygenated M9 buffer (pH 7.0).


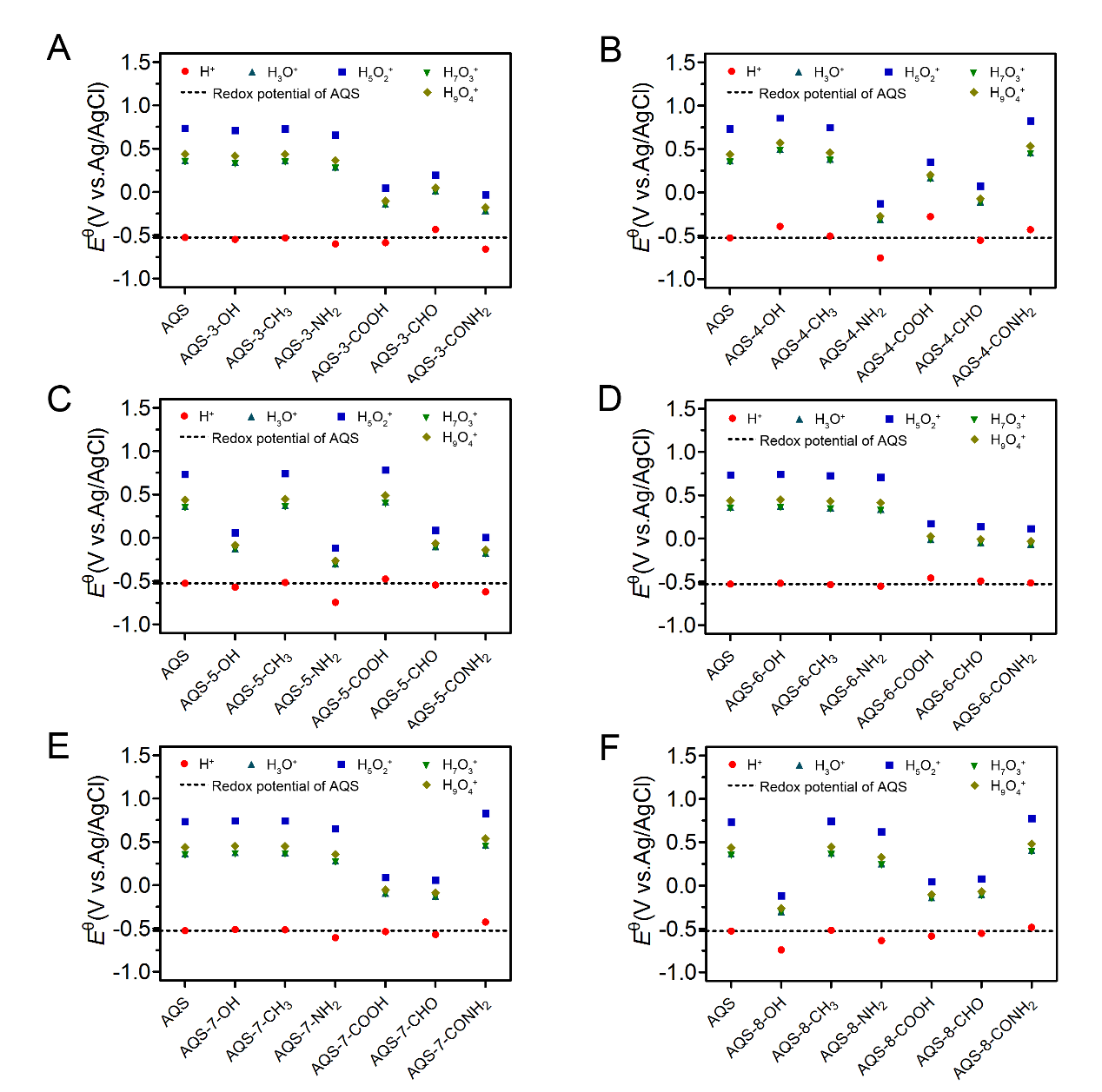


**Figure S4.** Redox potential in aqueous solution of AQS derivatives with different nature and position of the substituent groups. **(A)** Position 3. **(B)** Position 4. **(C)** Position 5. **(D)** Position 6. **(E)** Position 7. **(F)** Position 8.


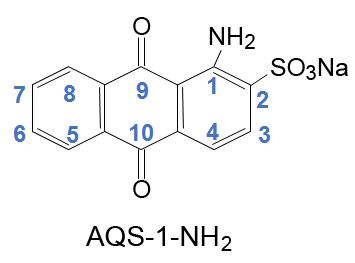

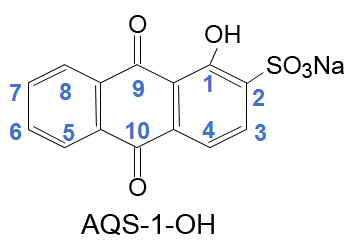


**Figure S5.** Molecular structures of AQS-1-NH_2_ and AQS-1-OH derivatives


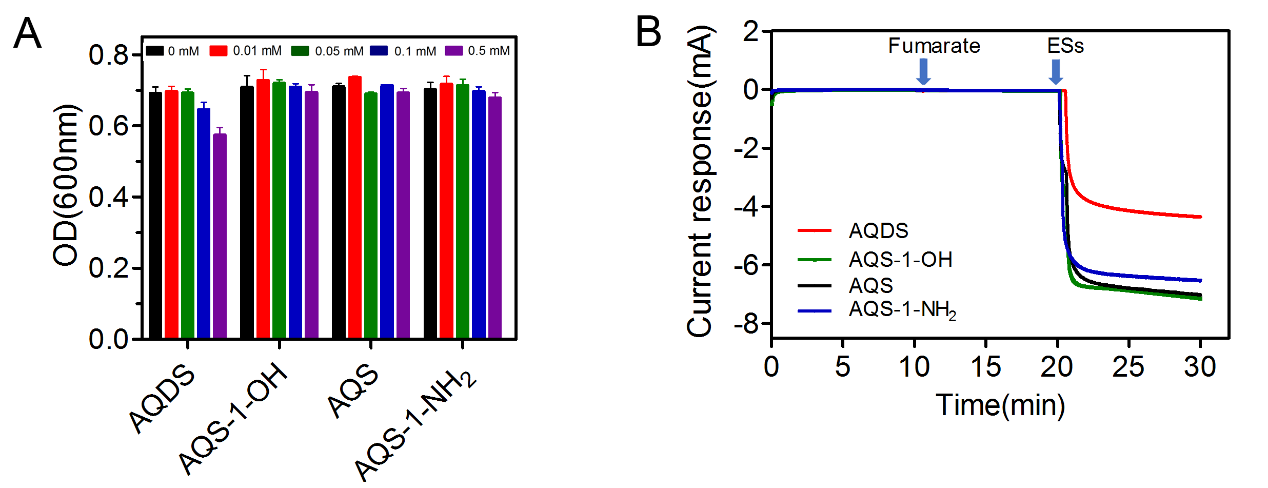


**Figure S6.** The cell toxicity assays of AQS and derivatives in MES.


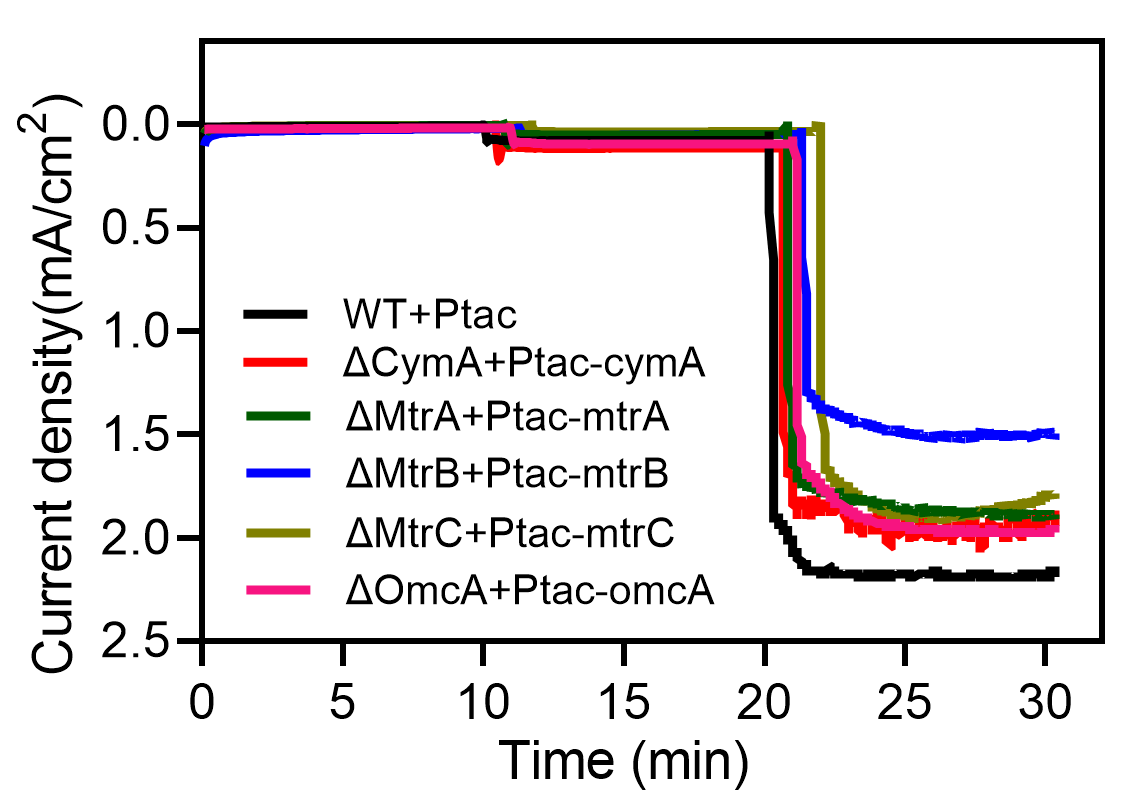


**Figure S7.** Current density assays in the corresponding complemented strains. The expression of genes under the control of Ptac promoter was induced by the addition of 0.1 mM isopropyl β- d-1-thiogalactopyranoside (IPTG). After a 4-hour induction, cells were harvested for electrochemical assay by the CHI1030C potentiostat.

**Supplementary tables:**

Table S1. Strains and plasmids used in this study

| Strain or plasmids | Description | Source |
| --- | --- | --- |
| *E. COLI STRAINS* | | |
| DH5α | *E. coli* derivative; competent cells for general cloning | Lab stock |
| WM3064 | *E. coli* derivative; donor strain for conjugation | 31 |
| *S. ONEIDENSIS STRAINS* | | |
| MR-1 | Representative wild-type *S. oneidensis* strain | 34 |
| *∆cymA* | *S. oneidensis* derivative; lacking the *cymA* gene | This work |
| *∆mtrA* | *S. oneidensis* derivative; lacking the *mtrA* gene | This work |
| *∆mtrB* | *S. oneidensis* derivative; lacking the *mtrB* gene | This work |
| *∆mtrC* | *S. oneidensis* derivative; lacking the *mtrC* gene | This work |
| *∆omcA* | *S. oneidensis* derivative; lacking the *omcA* gene | This work |
| *∆mtrC ∆omcA* | *S. oneidensis* derivative; lacking both the *mtrC* and *omcA* genes | This work |
| *PLASMIDS* | | |
| pHG1.0 | Gm^r^, Ap^r^; suicide vector | 31 |
| pHG1.01 | pHG1.0 contains *S. oneidensis cymA* flanking region | This work |
| pHG1.02 | pHG1.0 contains *S. oneidensis mtrA* flanking region | This work |
| pHG1.03 | pHG1.0 contains *S. oneidensis mtrB* flanking region | This work |
| pHG1.04 | pHG1.0 contains *S. oneidensis mtrC* flanking region | This work |
| pHG1.05 | pHG1.0 contains *S. oneidensis omcA* flanking region | This work |

Table S2. Primer sequences used in the study

| Primers for pHG1.01 construction | |
| --- | --- |
| CymA LF | CCAACTTGAGCTCGTAATCTGC |
| CymA LR | ACCTAAGATGTCATTTCTGCCACTC |
| CymA 5’O | GGGGACAAGTTTGTACAAAAAAGCAGGCTGGCATAGCTCTCACTATCGTCG |
| CymA 5’I | GGTCCGGGTTCGCTATCTATGCTAGGATGGAATATTTCGCGCT |
| CymA 3’I | ATAGATAGCGAACCCGGACCCGAGAAAGACCTGTGTGGATTGC |
| CymA 3’O | GGGGACCACTTTGTACAAGAAAGCTGGGTATCACATGACAAAGACGGCATTG |
| Primers for pHG1.02 construction | |
| MtrA LF | AGTATACCGGCATGAAAGCGG |
| MtrA LR | CACGTTTAAGTGAGAGTTCGAG |
| MtrA 5’O | GGGGACAAGTTTGTACAAAAAAGCAGGCTGAGCAACTATCACACACCCAAG |
| MtrA 5’I | GGTCCGGGTTCGCTATCTATGCCATAACTGCAGACATTGCC |
| MtrA 3’I | ATAGATAGCGAACCCGGACCGCTGCTTAAATTGCCATAGTCAG |
| MtrA 3’O | GGGGACCACTTTGTACAAGAAAGCTGGGTGCGCTATTGCTGTCGTAGGTAG |
| Primers for pHG1.03 construction | |
| MtrB LF | CACCAAGTACACGTCGCAAAAG |
| MtrB LR | TGCTCACGACTGACATTTAGCC |
| MtrB 5’O | GGGGACAAGTTTGTACAAAAAAGCAGGCTGTACACGTCGCAAAAGATCCTG |
| MtrB 5’I | GGTCCGGGTTCGCTATCTAT CAGTATTGGCATTCGCTAGACC |
| MtrB 3’I | ATAGATAGCGAACCCGGACCAGGACAATGACGCCGCAAAT |
| MtrB 3’O | GGGGACCACTTTGTACAAGAAAGCTGGGTCTTGGCTGCTCGCCTGTAAT |
| Primers for pHG1.04 construction | |
| MtrC LF | GTTCCAACGAGCTTTGCGTG |
| MtrC LR | GTGACAGTCGCTACAGGTCA |
| MtrC 5’O | GGGGACAAGTTTGTACAAAAAAGCAGGCTGGCGATATTAAGAACAATGGTGC |
| MtrC 5’I | GGTCCGGGTTCGCTATCTATCCGGTTAAGGCCATTGTGAC |
| MtrC 3’I | ATAGATAGCGAACCCGGACCGTTTCTACTGCCATACCCCAAC |
| MtrC 3’O | GGGGACCACTTTGTACAAGAAAGCTGGGTACAGGATCTTTTGCGACGTG |
| Primers for pHG1.05 construction | |
| OmcA LF | GCAATTAAACATCCATGGCGC |
| OmcA LR | ATCACTGGCATGTCGGCTTC |
| OmcA 5’O | GGGGACAAGTTTGTACAAAAAAGCAGGCTCTGGGAATATCGGTAATTGCG |
| OmcA 5’I | GGTCCGGGTTCGCTATCTATAGAAGGAGTGAAAGTAAACCGG |
| OmcA 3’I | ATAGATAGCGAACCCGGACCCATCTGAAAGCTGTGCAACG |
| OmcA 3’O | GGGGACCACTTTGTACAAGAAAGCTGGGTCTCACCACCATCACTACCAT |

Table S3. Standard Gibb’s free energy changes for the two electrons and one proton transfer reaction (2e^-^/1H^+^) of AQS

| AQS derivatives | ΔG^θ^ (2e^-^/1H^+^) (kcal/mol) | | | | |
| --- | --- | --- | --- | --- | --- |
|  | H^+^ | H_3_O^+^ | H_5_O_2_^+^ | H_7_O_3_^+^ | H_9_O_4_^+^ |
| AQS | -175.3784 | -204.3726 | -195.8749 | -195.5378 | -197.5996 |
| AQS-1-OH | -180.2791 | -209.2732 | -200.7755 | -200.4384 | -202.5002 |
| AQS-1-CH_3_ | -172.7784 | -201.7726 | -193.2748 | -192.9377 | -194.9996 |
| AQS-1-NH_2_ | -166.2163 | -195.2105 | -186.7127 | -186.3757 | -188.4375 |
| AQS-1-COOH | -176.1296 | -205.1238 | -196.6260 | -196.2889 | -198.3508 |
| AQS-1-CHO | -172.0037 | -200.9979 | -192.5002 | -192.1631 | -194.2249 |
| AQS-1-CONH_2_ | -179.5381 | -208.5323 | -200.0345 | -199.6974 | -201.7593 |
| AQS-3-OH | -172.0058 | -201.0000 | -192.5022 | -192.1651 | -194.2270 |
| AQS-3-CH_3_ | -173.7500 | -202.7441 | -194.2464 | -193.9093 | -195.9711 |
| AQS-3-NH_2_ | -166.8427 | -195.8369 | -187.3392 | -187.0021 | -189.0639 |
| AQS-3-COOH | -179.4843 | -208.4785 | -199.9807 | -199.6436 | -201.7055 |
| AQS-3-CHO | -186.4663 | -215.4605 | -206.9627 | -206.6256 | -208.6875 |
| AQS-3-CONH_2_ | -176.0661 | -205.0603 | -196.5625 | -196.2254 | -198.2873 |
| AQS-4-OH | -172.4023 | -201.3964 | -192.8987 | -192.5616 | -194.6234 |
| AQS-4-CH_3_ | -173.1245 | -202.1187 | -193.6210 | -193.2839 | -195.3457 |
| AQS-4-NH_2_ | -171.5437 | -200.5379 | -192.0401 | -191.7030 | -193.7649 |
| AQS-4-COOH | -193.5851 | -222.5793 | -214.0816 | -213.7445 | -215.8063 |
| AQS-4-CHO | -180.9140 | -209.9082 | -201.4104 | -201.0734 | -203.1352 |
| AQS-4-CONH_2_ | -175.2436 | -204.2378 | -195.7400 | -195.4029 | -197.4648 |
| AQS-5-OH | -180.2318 | -209.2260 | -200.7282 | -200.3911 | -202.4530 |
| AQS-5-CH_3_ | -173.2343 | -202.2285 | -193.7308 | -193.3937 | -195.4555 |
| AQS-5-NH_2_ | -172.1107 | -201.1048 | -192.6071 | -192.2700 | -194.3318 |
| AQS-5-COOH | -177.3162 | -206.3104 | -197.8126 | -197.4755 | -199.5374 |
| AQS-5-CHO | -181.3418 | -210.3360 | -201.8382 | -201.5011 | -203.5629 |
| AQS-5-CONH_2_ | -177.7330 | -206.7272 | -198.2294 | -197.8924 | -199.9542 |
| AQS-6-OH | -171.8210 | -200.8152 | -192.3175 | -191.9804 | -194.0422 |
| AQS-6-CH_3_ | -174.7695 | -203.7637 | -195.2659 | -194.9289 | -196.9907 |
| AQS-6-NH_2_ | -168.3897 | -197.3839 | -188.8861 | -188.5490 | -190.6109 |
| AQS-6-COOH | -185.5037 | -214.4979 | -206.0001 | -205.6630 | -207.7249 |
| AQS-6-CHO | -183.8933 | -212.8875 | -204.3897 | -204.0526 | -206.1145 |
| AQS-6-CONH_2_ | -182.8846 | -211.8788 | -203.3811 | -203.0440 | -205.1058 |
| AQS-7-OH | -174.0552 | -203.0494 | -194.5516 | -194.2145 | -196.2764 |
| AQS-7-CH_3_ | -174.3288 | -203.3230 | -194.8253 | -194.4882 | -196.5500 |
| AQS-7-NH_2_ | -168.9201 | -197.9143 | -189.4166 | -189.0795 | -191.1413 |
| AQS-7-COOH | -181.7313 | -210.7254 | -202.2277 | -201.8906 | -203.9524 |
| AQS-7-CHO | -180.1657 | -209.1598 | -200.6621 | -200.3250 | -202.3868 |
| AQS-7-CONH_2_ | -178.2823 | -207.2764 | -198.7787 | -198.4416 | -200.5034 |
| AQS-8-OH | -172.1926 | -201.1868 | -192.6891 | -192.3520 | -194.4138 |
| AQS-8-CH_3_ | -172.9810 | -201.9751 | -193.4774 | -193.1403 | -195.2021 |
| AQS-8-NH_2_ | -166.5251 | -195.5192 | -187.0215 | -186.6844 | -188.7462 |
| AQS-8-COOH | -179.5115 | -208.5056 | -200.0079 | -199.6708 | -201.7326 |
| AQS-8-CHO | -181.0209 | -210.0151 | -201.5174 | -201.1803 | -203.2421 |
| AQS-8-CONH_2_ | -176.0654 | -205.0596 | -196.5618 | -196.2247 | -198.2866 |

Table S4. Standard Gibb’s free energy changes for the two electrons and two protons transfer reaction (2e^-^/2H^+^) of AQS

| AQS derivatives | ΔG^θ^ (2e^-^/1H^+^) (kcal/mol) | | | | |
| --- | --- | --- | --- | --- | --- |
|  | H^+^ | H_3_O^+^ | H_5_O_2_^+^ | H_7_O_3_^+^ | H_9_O_4_^+^ |
| AQS | -182.2738 | -240.2621 | -223.2666 | -222.5924 | -226.7161 |
| AQS-1-OH | -190.3793 | -248.3677 | -231.3722 | -230.698 | -234.8217 |
| AQS-1-CH_3_ | -180.4415 | -238.4298 | -221.4343 | -220.7602 | -224.8838 |
| AQS-1-NH_2_ | -176.9375 | -234.9259 | -217.9304 | -217.2562 | -221.3799 |
| AQS-1-COOH | -182.068 | -240.0564 | -223.0609 | -222.3867 | -226.5104 |
| AQS-1-CHO | -182.4195 | -240.4078 | -223.4123 | -222.7382 | -226.8618 |
| AQS-1-CONH_2_ | -182.3596 | -240.3480 | -223.3525 | -222.6783 | -226.8020 |
| AQS-3-OH | -181.2337 | -239.222 | -222.2265 | -221.5524 | -225.676 |
| AQS-3-CH_3_ | -182.1022 | -240.0906 | -223.0951 | -222.4209 | -226.5446 |
| AQS-3-NH_2_ | -178.7869 | -236.7753 | -219.7798 | -219.1056 | -223.2293 |
| AQS-3-COOH | -184.4362 | -242.4245 | -225.429 | -224.7548 | -228.8785 |
| AQS-3-CHO | -187.3682 | -245.3565 | -228.3610 | -227.6869 | -231.8105 |
| AQS-3-CONH_2_ | -182.5868 | -240.5751 | -223.5796 | -222.9054 | -227.0291 |
| AQS-4-OH | -188.3908 | -246.3791 | -229.3836 | -228.7094 | -232.8331 |
| AQS-4-CH_3_ | -183.1439 | -241.1322 | -224.1367 | -223.4626 | -227.5862 |
| AQS-4-NH_2_ | -178.1863 | -236.1747 | -219.1792 | -218.505 | -222.6287 |
| AQS-4-COOH | -185.0498 | -243.0381 | -226.0427 | -225.3685 | -229.4921 |
| AQS-4-CHO | -182.1514 | -240.1397 | -223.1442 | -222.4700 | -226.5937 |
| AQS-4-CONH_2_ | -186.6311 | -244.6194 | -227.6239 | -226.9497 | -231.0734 |
| AQS-5-OH | -177.3126 | -235.301 | -218.3055 | -217.6313 | -221.755 |
| AQS-5-CH_3_ | -182.6864 | -240.6747 | -223.6792 | -223.0050 | -227.1287 |
| AQS-5-NH_2_ | -176.6162 | -234.6046 | -217.6091 | -216.9349 | -221.0586 |
| AQS-5-COOH | -184.5337 | -242.522 | -225.5265 | -224.8524 | -228.976 |
| AQS-5-CHO | -185.2028 | -243.1912 | -226.1957 | -225.5215 | -229.6452 |
| AQS-5-CONH_2_ | -183.8084 | -241.7967 | -224.8012 | -224.1270 | -228.2507 |
| AQS-6-OH | -182.706 | -240.6944 | -223.6989 | -223.0247 | -227.1484 |
| AQS-6-CH_3_ | -181.9453 | -239.9337 | -222.9382 | -222.2640 | -226.3877 |
| AQS-6-NH_2_ | -181.0732 | -239.0616 | -222.0661 | -221.3919 | -225.5156 |
| AQS-6-COOH | -187.0745 | -245.0628 | -228.0673 | -227.3932 | -231.5168 |
| AQS-6-CHO | -185.9292 | -243.9175 | -226.9220 | -226.2478 | -230.3715 |
| AQS-6-CONH_2_ | -187.7182 | -245.7066 | -228.7111 | -228.0369 | -232.1606 |
| AQS-7-OH | -182.873 | -240.8613 | -223.8658 | -223.1917 | -227.3153 |
| AQS-7-CH_3_ | -182.7816 | -240.7699 | -223.7744 | -223.1003 | -227.2239 |
| AQS-7-NH_2_ | -178.5053 | -236.4937 | -219.4982 | -218.824 | -222.9477 |
| AQS-7-COOH | -188.0814 | -246.0697 | -229.0742 | -228.4001 | -232.5237 |
| AQS-7-CHO | -186.0176 | -244.0060 | -227.0105 | -226.3363 | -230.4600 |
| AQS-7-CONH_2_ | -186.8297 | -244.8180 | -227.8225 | -227.1484 | -231.2720 |
| AQS-8-OH | -177.1869 | -235.1753 | -218.1798 | -217.5056 | -221.6293 |
| AQS-8-CH_3_ | -182.6890 | -240.6773 | -223.6818 | -223.0076 | -227.1313 |
| AQS-8-NH_2_ | -177.1395 | -235.1279 | -218.1324 | -217.4582 | -221.5819 |
| AQS-8-COOH | -185.07 | -243.0583 | -226.0628 | -225.3886 | -229.5123 |
| AQS-8-CHO | -185.3957 | -243.3841 | -226.3886 | -225.7144 | -229.8380 |
| AQS-8-CONH_2_ | -184.2425 | -242.2309 | -225.2354 | -224.5612 | -228.6849 |
